# Relationships between screen viewing and sleep quality for infants and toddlers in China: A cross-sectional study

**DOI:** 10.3389/fped.2022.987523

**Published:** 2022-10-10

**Authors:** Yumin Lin, Xueqin Zhang, Yinying Huang, Zhiwei Jia, Jing Chen, Wanling Hou, Lili Zhao, Guiyan Wang, Jiemin Zhu

**Affiliations:** ^1^ Department of Nursing, Women and Children's Hospital, School of Medicine, Xiamen University, Xiamen, China; ^2^ Department of Obstetrics and Gynecology, Women and Children's Hospital, School of Medicine, Xiamen University, Xiamen, China; ^3^ Department of Child Health, Women and Children's Hospital, School of Medicine, Xiamen University, Xiamen, China; ^4^ Department of Nursing, School of Medicine, Xiamen University, Xiamen, China

**Keywords:** screen viewing, sleep quality, infants, toddlers, China, health education

## Abstract

**Aim:**

Currently young children have more opportunity to access all kinds of media, while their sleep duration has been steadily decreasing. However, little is known about the relationships between screen viewing and sleep quality, and the reasons of screen viewing for children under three years old in China. This study aimed to describe the relationships between screen viewing and sleep quality of infants and toddlers in mainland China.

**Methods:**

A cross-sectional study was conducted. Eight hundred twenty-seven children were recruited at a health care unit from a university affiliated hospital in China, and the questionnaires were completed by their parents. An extended Brief Infant Sleep Questionnaire and a Screen Viewing Questionnaire were used to collect information on children's sleep quality and screen viewing. Multivariate linear regression models were used to assess the relationships between screen viewing and sleep quality among infants and toddlers, adjusted for sociodemographic variables.

**Results:**

Of the 827 children, 26.9% of the infants and 61.4% of the toddlers did not comply with the World Health Organization (WHO) guideline on screen time. Even after adjusting for the sociodemographic covariates for both infants and toddlers, negative relationships between screen time and total sleep time (*P *< 0.001), and screen time and nighttime sleep (*P *< 0.001) existed. TV viewing time was negatively related to infants' total sleep time (*β *=* *−0.15, *P *< 0.001) and toddlers' nighttime sleep (*β *=* *−0.1, *P *< 0.05). Smartphone viewing time was negatively related to toddlers' total sleep time (*β *=* *−0.12, *P *< 0.05) and daytime sleep (*β *=* *−0.22, *P *< 0.05). Parents who offered screen media for children when they needed to do house chores were more likely to report that their children had less total sleep time (*β *=* *−0.1, *P *< 0.05) and shorter longest sleep episode (*β *=* *−0.1, *P *< 0.05).

**Conclusion:**

The majority of toddlers did not meet the WHO guidelines on screen time in China. Screen time was negatively related to total sleep time and nighttime sleep among infants and toddlers. Practical strategies, such as education programs on children's screen viewing, more outdoor exercises and indoor parent-child activities, providing other educational materials instead of screening, early sleep, restricted use of TVs and smartphones, and screen co-viewing, are needed to improve young children's sleep quality and promote their development.

## Introduction

Recognizing the significance of sleep on young children's development (1, 2), WHO guidelines recommend that infants (≤12 months) should have 12–17 h of sleep and toddlers (13–36 months) should have 11–14 h of sleep (3). However, children's sleep duration has been steadily decreasing over recent decades, especially in Asian countries (4, 5). A survey in Beijing showed that only 29.5% of children meet the sleep guideline (6). Poor sleep problems are the most common reasons why parents ask help from health care workers in early childhood (7).

Lifestyle changes, especially increasing screen viewing, have been proven to cause shorter sleep duration among children (8). A systematic review, including 67 studies, found that screen time is negatively related with sleep duration and sleep timing in 90% of the studies on school-age children and adolescents (8). Li (2020) also did a systematic review and meta-analysis to explore the relationships between screen viewing and health outcomes among children under seven years old, and reported that children with excessive screen viewing would have more than twice the risk of shorter sleep duration. Moreover, the sleep of young children may negatively affect their further development (2). Sleep quality in early childhood has been reported to be associated with obesity (9), physical growth (10), motor development (11), and even cognitive development (12) and emotional regulation (13) in later life. Therefore, it is necessary to pay attention to the relationship between screen viewing and sleep in young children.

According to the latest WHO guideline on screen time (3), screen viewing is not recommended for infants and screen viewing should be less than 1 h for toddlers. However, currently young children have more opportunity to access all kinds of media (14). Half of the children less than one year old in America were daily screen watchers, and three-fourths of the children under four years old had their own mobile devices (15). Data from China showed that 25.6% of the children aged 18 months were found to engage in some screen time and 49.0% of the children aged 18–36 months were exposed to screen for more than one hour per day (16).

Although the reviews demonstrated the relationships between screen viewing and sleep quality, one review did not include data on children under three years old (8), and the other review only found four studies from Asian countries which target at children under three years old (1). Except for sleep duration, no other essential aspects of sleep quality, such as daytime sleep, nighttime sleep, and longest sleep episode, were available for meta-analysis because of the seldom report in original research (1, 17). At the same time, most studies provided data on TV viewing but did not investigate the contribution of other screen media, such as computers, mobile phones, and tablets, which are commonly used today (17, 18). In addition, little is known about the reasons for young children's screen viewing, as well as the effect of different kinds of screen viewing on sleep quality among children under three years old in China. This knowledge is vital to understanding how to decrease excessive screen viewing time and promote young children's sleep quality. To fill in the knowledge gaps, this study aimed to investigate the relationships between screen viewing (including common devices and reasons for screen viewing) and sleep quality (including total, daytime, nighttime sleep and longest sleep episode) for children under three-year-old in China. We hypothesized that screen time would be negatively related to total sleep time and the longest sleep episode for children under three years old. We also hypothesized that screen time spent on different electronic devices and different reasons for screen viewing would be related to sleep quality.

## Methods

### Design

A cross-sectional design.

### Setting and participants

The inclusion criteria were children who (1) were 0–3 years old, (2) were born full-term in a singleton pregnancy (37–42 weeks of gestation; ≥2500 g birth weight), (3) had no history of cognitive or behavioral deficits, (4) had no complications or congenital malformations, and (5) had no multiple or prolonged hospitalizations (>5 days). We excluded children whose parents were illiterate in Mandarin.

Participants were recruited from a university affiliated hospital in Xiamen, China. This hospital has a department of child health, where more than 30,000 children do their routine physical examination per year.

### Sample size calculation

RaoSoft (http://www.raosoft.com/samplesize.html) was used to calculate the sample size. The estimated total number of children under three years old in Xiamen is approximately 190,000 (19). With a margin error at 5%, a confidence level at 95%, and a response rate at 50%, a minimum sample size of 384 was required in this survey.

### Outcomes and measurement

A sociodemographic questionnaire was self-developed by the research team. Data on the children's age, sex, BMI, daily outdoor exercise time, primary caregivers, and annual family income were collected.

Screen viewing was assessed using the Screen Viewing Questionnaire (SVQ), which was developed by our research team on the basis of the literature review (20, 21). Parents were required to answer the following question: “How long did your child spend on the following devices per day for the last month?” Devices included TVs, smartphones, computers, and others (e.g., tablets, early education machines, and game consoles). For each device, participants were asked to select the options None, <0.5, 0.5–1, 1–2, 2–3, 3–4, 4–5, and ≥5 h per day. The above options were converted to the average screen time as 0, 0.25, 0.75, 1.5, 2.5, 3.5, 4.5, and 5 h, respectively, to generate an approximately continuous variable of screen time per day (22, 23). Total screen time was the sum of the screen time spent on all the devices. If children have screen time, their parents were asked to tick the following multiple choices questions to explore the corresponding reasons for screen view including education, managing tantrums, entertainment, and for parents to do household chores (24). Ten experts were invited to evaluate the content validity, resulting in a total item-level content validity index (I-CVI) of 0.8 and a scale-level content validity index, averaging method (S-CVI/ave) of 0.97.

Children's sleep quality was measured using the Chinese version of the extended Brief Infant Sleep Questionnaire (BISQ-E) (25). BISQ-E is well validated for assessing the average sleep patterns of children aged 0–3 years (26). The questionnaire was translated into Chinese and was widely used in many cross-cultural studies (27). Participants were required to complete the BISQ-E on the basis of their children's sleep patterns and sleep-related behaviors over the past two weeks. In our study, we only chose total sleep time (the primary outcome), daytime sleep, nighttime sleep, and longest sleep episode as sleep variables, which are clinically significant for assessing sleep quantity and quality in young children (28).

### Data collection

After receiving ethical approval from the hospital, the head nurse in the Unit of Child Health Care was informed of the aim of the study, as well as the inclusion and exclusion criteria. Convenience sampling was used to collect data for this study. The head nurse approached 935 eligible children's caregivers when they took their children to the hospital for routine physical examinations from March to December 2020. Prior to data collection, each participant received an information sheet about the purpose, content, and significance of the study. After signing the consent form, interested caregivers took 10–15 min to complete the questionnaires. Participants who had multiple children were asked to only report their youngest child's screen review and quality of sleep. Researchers checked the integrity of the questionnaires immediately to make sure that all the questions were answered.

### Ethical consideration

This study was approved by the Ethics Committee of the participating hospital (Ethical Approval NO.KY-2020-109). Participation in our study was voluntary and anonymous. Written informed parental consent was obtained for participation in the study. Data about the identity of participants were not disclosed at any stage of the study.

### Data analysis

Data were verified and analyzed using IBM SPSS 26.0 software (IBM Corp, 2019). The sociodemographic characteristics of the participants and their families were presented with means (with standard deviation) for continuous variables and frequencies (with percentages) for categorical variables. We classified our participants into infants and toddlers, and the total screen time was divided into 0, >0 ∼≤1 h, and >1 h. The two independent sample *t*-test was used to investigate the differences in sleep quality between children with or without screen viewing. Multivariate linear regression models were used to assess the relationships between screen viewing (including total screen time, screen time on different devices, and reasons for screen viewing) and sleep quality (including total sleep time, daytime sleep, nighttime sleep, and longest sleep episode). Each screen viewing variable and sleep variable were entered into a separate model for both infants and toddlers, resulting in 48 multivariate linear regression models. Each model was adjusted for children's sex, age, daily outdoor exercise time, primary caregivers, BMI, and family annual income. Differences were considered statistically significant with a *P*-value < 0.05.

## Results

Of the 915 eligible children's caregivers approached, 45 caregivers refused as they had no time, with a response rate of 95.08%. Of the 870 completed questionnaires, 34 participants reported children's total sleep time to be less than 4 h, and 9 participants reported children's total sleep time to be more than 22 h. According to the author of BISQ-E, total sleep time less than 4 h or more than 22 h were considered inappropriate or extreme data, thus being excluded (25). Therefore, these participants were excluded, resulting in an analytic sample of 827 participants.

### Characteristics of the study population

Of the total population, 827 children were included, comprising 361 infants (43.7%) and 466 toddlers (56.3%). The mean age of the children in this study was 17.3 months (SD = 11.8). There were 471 girls (57%) and 356 boys (43%). Children's BMI was 16.1 (SD = 2.6), and children's daily outdoor exercise time was 2.2 h (SD = 1.5). Most of the children's primary caregivers were mothers (*n* = 471, 57%), followed by grandparents (*n* = 253, 30.6%). Parents reported an annual family income of more than 26,000 dollars (36.3%), 17,000–26,000 dollars (36.3%), and less than 17,000 dollars (27.4%). The characteristics of infants and toddlers and their families are summarized in Table 1.

**Table 1:** Sociodemographic data of participants (*n* = 827).

	Overall		Infants (*n* = 361, 43.7%)		Toddlers (*n* = 466, 56.3%)	
**Characteristics**	Mean (SD)	*n* (%)	Mean (SD)	*n* (%)	Mean (SD)	*n* (%)
**Children's age (mos)**	17.3 (11.8)		5.8 (3.8)		26.2 (7.4)	
**Children's gender**						
Girl		471 (57)		218 (60.4)		253 (54.3)
Boy		356 (43)		143 (39.6)		213 (45.7)
**BMI**	16.1 (2.6)		16.5 (3)		15.8 (2.2)	
**Children's daily outdoor exercise time (hrs)**	2.2 (1.5)		1.8 (1.4)		2.5 (1.5)	
**Primary caregiver**						
Mother		471 (57)		271 (75.1)		200 (42.9)
Father		50 (6)		7 (1.9)		43 (9.2)
Grandparents		253 (30.6)		68 (18.8)		185 (39.7)
Others^a^		53 (6.4)		15 (4.2)		38 (8.2)
**Annual family income ($)**						
<17,000		227 (27.4)		115 (31.9)		112 (24)
17,000–26,000		300 (36.3)		134 (37.1)		166 (35.6)
>26,000		300 (36.3)		112 (31)		188 (40.3)
**Screen viewing**						
**Total screen time**						
No viewing		313 (37.8)		264 (73.1)		49 (10.5)
>0 ∼≤1 h		205 (24.8)		74 (20.5)		131 (28.1)
>1 h		309 (37.4)		23 (6.4)		286 (61.4)
**Screen time on different devices (hrs)**						
TV	0.6 (0.9)		0.1 (0.4)		0.9 (0.9)	
Smartphone	0.3 (0.6)		0.1 (0.3)		0.5 (0.8)	
Computer	0.1 (0.6)		0.0 (0.2)		0.2 (0.7)	
Others^b^	0.1 (0.5)		0.0 (0.1)		0.2 (0.6)	
**Reasons for screen viewing**^c^						
Educational		198 (23.9)		39 (10.8)		159 (34.1)
Management of tantrums		148 (17.9)		20 (5.5)		128 (27.5)
Entertainment		127 (15.4)		16 (4.4)		111 (23.8)
Allowing parents to do household chores		127 (15.4)		27 (7.5)		100 (21.5)
**Sleep variable (hrs)**						
Total sleep time	12.2 (2.4)		13.8 (2.5)		10.9 (1.4)	
Nighttime sleep	8.9 (1.3)		9.2 (1.5)		8.7 (1.1)	
Daytime sleep	3.2 (1.9)		4.6 (2.1)		2.2 (0.8)	
Longest sleep episode	6.7 (2.4)		5.9 (2.6)		7.4 (2.1)	

Note. hrs, hours; mos, months; yrs, years.

^a^ Includes baby-sitter or relatives who help take care of children.

^b^ Includes iPad, tablet, game consoles, or similar devices.

^c^ Multiple choices, caregivers need to answer the question when they reported their children's exposure to screen.

### Screen viewing

Of the total number of children, 313 (37.8%) reported no screen time, 205 (24.8%) reported less than 1 h per day of screen time, and 309 (37.4%) reported more than 1 h per day of screen time. In our study, 97 (26.9%) infants and 286 (61.4%) toddlers did not comply with the latest WHO guideline on screen time with no screen viewing for infants and no more than 1 h of screen time for toddlers. TVs and smartphones are the two most common devices used by children. The mean time children spent on TVs and smartphones was 0.6 (SD = 0.9) hours and 0.3 (SD = 0.6) hours, respectively. Reasons for screen viewing were education (198, 23.9%), management of tantrums (148, 17.9%), entertainment (127, 15.4%), and allowing parents to do household chores (127, 15.4%) (Table 1).

### Sleep quality

The total sleep time for all children was 12.2 (SD = 2.4) hours. Mean nighttime and daytime sleep was 8.9 (SD = 1.3) hours and 3.2 (SD = 1.9) hours. The longest sleep episode was 6.7 (SD = 2.4) hours. Detailed information on the sleep time of infants and toddlers is shown in Table 1.

### Sleep quality differences between children with or without screen viewing

Compared to infants with screen viewing, infants without screen viewing had more total sleep time, daytime sleep, and nighttime sleep (*P* < 0.001). No significant difference was found in longest sleep episode in infant group (*P* = 0.62). Compared to toddlers with screen viewing, toddlers without screen viewing had more total sleep time, nighttime sleep and longest sleep episode without screen viewing (*P* < 0.001). No significant difference was found in daytime sleep in toddler group (*P* = 0.08) (Table 2).

**Table 2:** Comparison of sleep quality between children with or without screen viewing.

	Infants (*n* = 361)			Toddlers (*n* = 466)		
	Screen viewing (*n* = 264)	No screen viewing (*n* = 97)	*P*	Screen viewing (*n* = 417)	No screen viewing (*n* = 49)	*P*
Total sleep time	11.87 ± 1.76	14.50 ± 2.39	<0.001	10.82 ± 1.38	11.88 ± 1.18	<0.001
Daytime sleep	3.39 ± 1.4	4.98 ± 2.22	<0.001	2.24 ± 0.81	2.03 ± 0.68	0.08
Nighttime sleep	8.48 ± 1.17	9.52 ± 1.52	<0.001	8.57 ± 1.08	9.86 ± 0.91	<0.001
Longest sleep episode	5.79 ± 2.19	5.93 ± 2.70	0.62	7.25 ± 2.07	8.27 ± 2.22	<0.001

Note: The two independent sample *t*-test was used.

### Relationships between screen viewing and sleep quality among infants

Spending a total screen time of more than 1 h among infants was related to a decrease in total sleep time (*β* = −0.2, adjusted *R*^2 ^= 0.35, *P *< 0.001), daytime sleep (*β* = −0.1, adjusted *R*^2 ^= 0.39, *P *< 0.001), and nighttime sleep (*β* = −0.2, adjusted *R*^2 ^= 0.1, *P *< 0.001). Spending a total screen time of 0–1 h among infants was related to a decrease in total sleep time (*β* = −0.3, adjusted *R*^2 ^= 0.35, *P *< 0.001) and nighttime sleep (*β*^ ^=^ ^−0.3, adjusted *R*^2 ^= 0.1, *P *< 0.001). Spending more time on watching TV, but not other devices, was related to decreased total sleep time (*β*^ ^=^ ^−0.15, adjusted *R*^2 ^= 0.3, *P *< 0.001). See the details in Table 3. No significant relationship was found between the reasons for screen viewing and sleep variables.

**Table 3:** Relationships between screen viewing and sleep outcome among infants (*n* = 361).

** **	**Total sleep time**			**Daytime sleep**			**Nighttime sleep**			**Longest sleep episode**		
	**B (SE)**	**Beta**	**95%CI**	**B (SE)**	**Beta**	**95%CI**	**B (SE)**	**Beta**	**95%CI**	**B (SE)**	**Beta**	**95%CI**
**Total screen time**												
No viewing	REF											
>0 ∼≤1 h	**−1.6** (**0.3)**	**−0**.**3**	(**−2.2 to −1.1)**	**−**0.4 (0.2)	**−**0.1	(**−**0.8 to 0.1)	**−1.3** (**0.2)**	**−0**.**3**	**(−1.7 to −0.9)**	**−**0.6 (0.4)	**−**0.1	(**−**1.3 to 0.1)
>1 h	**−2.1** (**0.5)**	**−0**.**2**	(**−3 to −1.2)**	**−0.8** (**0.4)**	**−0**.**1**	**(−1.6 to −0.1)**	**−1.3** (**0.3)**	**−0**.**2**	**(−1.9 to −0.6)**	**−**0.9 (0.6)	**−**0.1	(**−**2.1 to 0.2)
**Adjusted *R*^2^**	**0.35**			**0**.**39**			**0.1**			**0.07**		
**Screen time on different devices (hrs)**												
TV	**−0.84** (**0.29)**	**−0**.**15**	(**−1.41 to −0.27)**	**−**0.35 (0.23)	**−**0.07	(**−**0.81 to 0.11)	**−**0.49 (0.21)	**−**0.14	(**−**0.9 to **−**0.09)	**−**0.43 (0.34)	**−**0.07	(**−**1.1 to 0.25)
Smartphone	**−**0.33 (0.5)	**−**0.04	(**−**1.3 to 0.65)	**−**0.13 (0.4)	**−**0.02	(**−**0.92 to 0.66)	**−**0.2 (0.35)	**−**0.04	(**−**0.89 to 0.5)	**−**0.29 (0.59)	**−**0.03	(**−**1.45 to 0.87)
Computer	**−**0.36 (0.74)	**−**0.03	(**−**1.81 to 1.09)	**−**0.23 (0.59)	**−**0.02	(**−**1.39 to 0.94)	**−**0.13 (0.52)	**−**0.02	(**−**1.16 to 0.9)	**−**1.49 (0.87)	**−**0.11	(**−**3.2 to 0.22)
Others	**−**1.03 (1.11)	**−**0.06	(**−**3.21 to 1.14)	**−**0.3 (0.89)	**−**0.02	(**−**2.05 to 1.45)	**−**0.73 (0.79)	**−**0.07	(**−**2.28 to 0.82)	1.67 (1.31)	0.09	(**−**0.9 to 4.24)
**Adjusted *R*^2^**	**0.3**			**0**.**38**			0.02			**0.07**		

Note. Multivariate linear regression models were used to assess the relationships between screen viewing and sleep quality. hrs, hours; B, unstandardized regression coefficient; SE, standard error of regression coefficient; Beta, standardized regression coefficient. 95% CI, 95% bias corrected and accelerated confidence intervals of unstandardized regression coefficient as estimated by means of bootstrapping. Significant regression coefficients (*P* < 0.05) are printed in bold. Adjusted for children's sex, age, daily outdoor exercise time, primary caregivers, BMI, and family annual income.

### Relationships between screen viewing and sleep quality among toddlers

Spending a total screen time of more than 1 h among toddlers was related to a decrease in total sleep time (*β*=-0.38, adjusted *R*^2 ^= 0.08, *P* < 0.001), nighttime sleep (*β*^ ^=^ ^−0.58, adjusted *R*^2 ^= 0.13, *P *< 0.001), and longest sleep episode (*β* = −0.35, adjusted *R*^2 ^= 0.04, *P *< 0.001). However, spending a total screen time of more than 1 h was related to an increase in daytime sleep (*β* = 0.17, adjusted *R*^2 ^= 0.04, *P *< 0.05). Similarly, spending a total screen time of 0–1 h among toddlers was related to a decrease in total sleep time, nighttime sleep, and longest sleep episode and an increase in daytime sleep. In addition, spending more time on TV was related to less nighttime sleep (*β* = −0.1, adjusted *R*^2 ^= 0.03, *P *< 0.05). Spending more time on a smartphone was related to a decrease in total sleep time (*β* = −0.12, adjusted *R*^2 ^= 0.04, *P *< 0.05) and daytime sleep (*β* = −0.11, adjusted *R*^2 ^= 0.04, *P *< 0.05). Parents who offered screen media for children when they needed to do house chores were more likely to report that their children had less total sleep time (*β* = −0.1, adjusted *R*^2 ^= 0.03, *P *< 0.05) and shorter longest sleep episode (*β* = −0.1, adjusted *R*^2 ^= 0.02, *P *< 0.05) (see Table 4). No significant relationship was found between other reasons for screen viewing and sleep variables.

**Table 4:** Relationships between screen viewing and sleep outcome among toddlers (*n* = 466).

** **	**Total sleep time**			**Daytime sleep**			**Nighttime sleep**			**Longest sleep episode**		
	**B (SE)**	**Beta**	**95%CI**	**B (SE)**	**Beta**	**95%CI**	**B (SE)**	**Beta**	**95%CI**	**B (SE)**	**Beta**	**95%CI**
**Total screen time**	** **											
No viewing	REF											
>0 ∼≤1 h	**−0.7** (**0.23)**	**−0**.**22**	**(−1.15 to −0.25)**	**0.3** (**0.13)**	**0**.**17**	**(0.04 to 0.56)**	**−1** (**0.18)**	**−0**.**4**	**(−1.35 to −0.64)**	**−0.99** (**0.35)**	**−0**.**21**	**(−1.68 to -0.31)**
>1 h	**−1.08** (**0.22)**	**-0**.**38**	**(−1.52 to −0.65)**	**0.27** (**0.13)**	**0**.**17**	**(0.02 to 0.52)**	**−1.35** (**0.17)**	**−0**.**58**	**(−1.69 to −1.02)**	**−1.5** (**0.34)**	**−0**.**35**	**(−2.16 to −0.84)**
**Adjusted *R*^2^**	**0.08**			**0**.**04**			**0.13**			**0.04**		
**Screen time on different devices (hrs)**												
TV	**−**0.07 (0.07)	**−**0.05	(**−**0.21 to 0.07)	0.05 (0.04)	0.06	(**−**0.03 to 0.14)	**−0.12** (**0.06)**	**−0**.**1**	**(−0.24 to −0.01)**	0.01 (0.11)	0.004	(**−**0.21 to 0.23)
Smartphone	**−0.22** (**0.1)**	**−0**.**12**	**(−0.41 to −0.03)**	**−0.11** (**0.05)**	**−0**.**1**	**(−0.22 to 0)**	**−**0.11 (0.08)	**−**0.07	(**−**0.26 to 0.04)	**−**0.37 (0.15)	**−**0.13	(**−**0.66 to **−**0.09)
Computer	**−**0.14 (0.1)	**−**0.07	(**−**0.35 to 0.06)	**−**0.05 (0.06)	**−**0.05	(**−**0.17 to 0.06)	**−**0.09 (0.08)	**−**0.06	(**−**0.26 to 0.07)	**−**0.01 (0.16)	**−**0.004	(**−**0.32 to 0.3)
Others	0.12 (0.13)	0.05	(**−**0.13 to 0.37)	0.1 (0.07)	0.08	(**−**0.04 to 0.24)	0.02 (0.1)	0.01	(**−**0.18 to 0.22)	0.1 (0.19)	0.03	(**−**0.28 to 0.48)
**Adjusted *R*^2^**	**0.04**			0.04			**0**.**03**			**0.01**		
**Reason for screen viewing** Allowing parents to do household chores^a^	**−0.33** (**0.16)**	**−0**.**1**	**(−0.64 to −0.02)**	**−**0.07 (0.09)	**−**0.04	(**−**0.25 to 0.11)	**−**0.26 (0.13)	**−**0.1	(**−**0.51 to **−**0.02)	**−0.48** (**0.24)**	**−0**.**1**	**(−0.94 to −0.01)**
** Adjusted *R*^2^**	**0**.**03**			**0**.**05**			0.01			**0**.**02**		

Note. Multivariate linear regression models were used to assess the relationships between screen viewing and sleep quality. hrs, hours; B, unstandardized regression coefficient; SE, standard error of regression coefficient; Beta, standardized regression coefficient. 95% CI, 95% bias corrected and accelerated confidence intervals of unstandardized regression coefficient as estimated by means of bootstrapping. Significant regression coefficients (*P* < 0.05) are printed in bold. Adjusted for children's sex, age, daily outdoor exercise time, primary caregiver, BMI and family annual income.

^a^ The reference group reported reasons for screen exposure are not for parents to do household chores.

## Discussion

Our study was the first to investigate the relationships between screen viewing and sleep quality among infants and toddlers in mainland China. In our study, after adjusting for children's sex, age, daily outdoor exercise time, primary caregivers, BMI, and annual family income, total screen time was still negatively related to total and nighttime sleep for infants and toddlers.

In our study, 26.9% of the infants and 61.4% of the toddlers did not comply with the latest WHO guideline on screen time (3). A review included 63 studies with 89,163 participants to explore the global prevalence of children's screen time (29). This review concluded that approximately 75.3% children younger than 2 years old and 64.4% of the children aged 2–5 years did not meet the screen time guideline (29). Our study highlighted the need to offer corresponding education and resources to parents to fit the guideline recommendations into their lives.

Our study found a negative relationship between screen time and total and nighttime sleep for both infant and toddlers, as well as the negative relationship between screen time and daytime sleep for infants. This finding is supported by previous literature (17, 30–32). Unlike adults, daytime sleep is an important part of infants’ sleep. A cross-cultural study of infants from 17 countries found that children have an average of more than 2 h of daytime sleep (33). Study showed daytime sleep during infancy had a positive impact on infant's memory and learning (34). Increased screen time increases children's sedentary time, which reduces their outdoor exercise time, thus affecting their sleep quality (1). Moreover, the sleep-wake cycle may be interrupted by a decrease in the hormone melatonin, which can be affected by exposure to screen bright light before bedtime (30). Thus, various strategies should be applied to reduce screen time, such as more outdoor exercises and indoor parent-child activities, eventually promoting total and nighttime sleep.

In our study, more screen time was related to a shorter longest sleep episode but a longer daytime sleep for toddlers. Few studies examined the relationships between screen viewing and daytime sleep and longest sleep episode (35). In China, children always do not attend kindergarten until three years old. Therefore, toddlers in China may have time to compensate for losing nighttime sleep in daytime since they do not need to wake up as school requires (36). However, the total hormonal production, especially the growth hormone, reaches its peak between 22:00–2:00 and 5:00–7:00 (37). Thus, a variety of strategies should be carried out to reduce screen time in the evening to go early to bed.

In our study, TVs and smartphones were the two most common screen devices used by young children. Viewing of different screen devices was related to the sleep quality of children with different ages. TV viewing time was negatively related to infants' total sleep time and toddlers' nighttime sleep. Smartphone viewing time was negatively related to toddlers' total and daytime sleep. No relationship between screen viewing on computers or other electronic devices and sleep quality was found. These results were similar to those of existing research on preschool children (38). However, another study on school-age children reported that no specific type of screen time resulted in significantly shorter total sleep time than another (39). The underlying reason may be related to different ages (16). In our study, children under three years old spent little time on computers or other screen devices. Thus, it was difficult to detect a significant relationship between screen time spent on other electronic devices and sleep quality. Therefore, different targeted strategies need to be developed for children with different ages, such as reducing TV viewing time for infants and toddlers and decreasing smartphone viewing time for toddlers.

In our study, education and management of tantrums were the two most common reasons for screen viewing. This is different from studies in western countries, which showed that most parents (65%–70%) let their children view screens because of doing chores (15, 40). The reasons for screen viewing during early childhood could vary substantially according to culture and context. Asian parents have a more positive belief that screen viewing is educational and useful for their children (41). However, the educational effect of screen viewing is still controversial (42). Parents' highly positive belief in screen viewing results in more screen viewing for children (43). Thus, education programs are needed to alter parents’ attitude toward screen viewing (44). Examining cross-cultural differences in the reasons for screen viewing can provide more culture-sensitive strategies to reduce screen viewing time effectively in infancy and toddlerhood in different countries. For example, due to high expectations of children's educational performance among Chinese parents, they utilize educational apps to start their children's early learning. Thus, providing other educational materials instead of screening may be an effective strategy to reduce screen viewing for young Chinese children (45).

In our study, toddlers' parents who offered screen media to children in order to do house chores were more likely to report that their children had shorter longest sleep episodes. Sometimes parents use screen media as a “babysitter” (46). Parents use media to make children busy when they are doing household chores, which is a common phenomenon, especially during the COVID-19 pandemic (47). Leaving children alone to screen may cause several problems, including unknown media content and decreased parent-child interaction. Previous studies stated that co-viewing increases parent-child interaction, ensures the health and safety of screen content, and helps children better understand the content (14, 42). Whether screen viewing may benefit children largely depends on appropriate media content (48, 49) and co-viewing with parents (50, 51). Further interventions should not only focus on reducing screen viewing time but also set rules (such as co-viewing and appropriate screen content) for screen viewing to gain the potential benefits of screen viewing.

This study also has some limitations. First, this is a cross-sectional study, and the causal relationships between screen viewing and sleep quality in children under three years old are not established. Second, we recruited our participants from only one university affiliated hospital, which may limit the generalization of our results. Third, some clinical important parameters such as night time awakenings and settling difficulties have not been included. Such information may help interpret the findings. Fourth, key screen time related variables such as quality of the content and nature of viewing (solitary/ co viewing) has not been assessed, which is essential for formulating recommendations. Future studies with more robust design and more comprehensive parameters are warranted to further explore such relationships, thus help better improve children's screen view and sleep quality.

## Conclusion

This study showed that the majority of toddlers did not meet the WHO guideline on screen time in mainland China. More screen time was related to less total and nighttime sleep for infants and toddlers. TV viewing time was negatively related to infants' total sleep time and toddlers' nighttime sleep. Smartphone viewing time was negatively related to toddlers' total and daytime sleep. Such as education programs on children's screen viewing, providing other educational materials instead of screening, more outdoor exercises and indoor parent-child activities, early sleep, restricted use of TVs and smartphones, and screen co-viewing, are needed to improve young children's sleep quality and promote their development.

## Data Availability

The raw data supporting the conclusions of this article will be made available by the authors, without undue reservation.
